# Relationship Between Mental Health and Health-Related Quality of Life of Orphans of Femicide

**DOI:** 10.3390/children13081021

**Published:** 2026-07-31

**Authors:** Erick Fontenele, Cristiane Faiad, Ana Isabel Sani

**Affiliations:** 1 Graduate Program in Clinical Psychology and Culture, Institute of Psychology, University of Brasília, Brasília 70710-907, Brazil; erickgfontenele@gmail.com (E.F.); crisfaiad@gmail.com (C.F.); 2 Centre for Research in Social Technologies (CEPATS), University of Brasília, Brasília 70710-907, Brazil; 3 Faculty of Human and Social Sciences, University Fernando Pessoa (UFP), Praça 9 de Abril, 349, 4249-004 Porto, Portugal; 4 Research Centre on Child Studies (CIEC), University of Minho, 4710-057 Braga, Portugal

**Keywords:** orphaned children of femicide, health-related quality of life, depression, anxiety

## Abstract

**Highlights:**

**What are the main findings?**

- Higher depression levels were linked to poorer health-related quality of life.
- Depression was a stronger predictor of quality of life than anxiety.
- Depression explained much of the link between anxiety and quality of life.

**What are the implications of the main findings?**

- Early detection of depression may improve quality of life.
- Mental health support should address the needs of adolescents and girls.
- Long-term psychosocial support is important after femicide-related orphanhood.

**Abstract:**

**Background/Objectives:** Femicide-related orphanhood exposes children and adolescents to psychological distress and may disrupt their health-related quality of life (HRQoL). This study examined the relationships between anxiety, depression, and HRQoL among children and adolescents orphaned by femicide and tested whether depressive symptoms mediate the association between anxiety and HRQoL. **Methods:** A cross-sectional study was conducted with 57 children and adolescents aged 8 to 18 years who had been orphaned by femicide and were residing in the Federal District of Brazil. Participants completed the Revised Child Anxiety and Depression Scale (RCADS-25) to assess anxiety and depressive symptoms and the KIDSCREEN-27 to evaluate HRQoL. Data were analyzed using Spearman correlation coefficients, linear regression models with bootstrap procedures, and mediation analyses to explore direct and indirect relationships among variables. **Results:** Depressive symptoms were negatively associated with HRQoL, while anxiety symptoms showed a weaker negative association with HRQoL. In the regression analysis, depression emerged as the only significant predictor of HRQoL. Mediation analysis indicated that the relationship between anxiety and HRQoL was largely explained by depressive symptoms, suggesting a partial indirect effect of anxiety through depression. **Conclusions:** The findings indicate that depressive symptoms may play a central role in the relationship between anxiety and perceived quality of life among children and adolescents orphaned by femicide. These results highlight the importance of assessing both anxiety and depression in this population and suggest that interventions addressing depressive symptomatology may contribute to improving psychosocial well-being and HRQoL following exposure to severe family violence.

## 1. Introduction

Femicide is one of the most extreme forms of gender-based violence and a serious public health, human rights, and social justice [1,2,3]. Beyond the loss of a woman’s life, femicide generates profound, long-term, and intergenerational consequences that extend to families, communities, and social protection systems [3,4]. Among those most severely affected are children and adolescents who lose their mothers as a result of lethal violence, frequently perpetrated by intimate partners or other individuals within the victim’s close social circle. As a result, these children are often exposed to heightened vulnerability and social invisibility [3,5,6].

In Brazil, more than 1400 femicides were recorded in 2024, with most cases occurring in the context of domestic violence and involving intimate partners as perpetrators [7]. Because many of these women were mothers, the number of children and adolescents affected by femicide-related orphanhood continues to grow. Unlike other forms of parental loss, orphanhood resulting from femicide is marked not only by the sudden death of the mother but also by a range of traumatic and destabilizing experiences, including exposure to violence, family disruption, abrupt changes in caregiving arrangements, and, in many cases, the removal or incarceration of the father when he is responsible for the crime [1,3].

Orphanhood resulting from femicide differs qualitatively from other forms of parental loss: it involves not only the sudden death of the mother but also traumatic rupture of attachments, direct or indirect exposure to violence, interaction with the criminal-justice system, and often the simultaneous loss of the father or mother’s partner—who may be the perpetrator and be subsequently imprisoned, commit suicide, or withdraw from the family [3,6]. Furthermore, these children and adolescents often face abrupt changes in residence, school, and caregivers—frequently being placed with grandparents, uncles, or other relatives—which exacerbates family instability and weakens support networks [1,8]. Together, these experiences create a context of cumulative adversity characterized by emotional loss, disrupted attachments, insecurity, and significant psychosocial challenges. Exposure to these conditions may negatively affect emotional well-being, social functioning, and cognitive development, with consequences that can persist throughout childhood and adolescence [3,9,10].

Studies have shown that children orphaned by lethal violence are at increased risk of developing mental health problems, including symptoms of anxiety, depression, behavioral difficulties, attachment disturbances, and traumatic distress [3,5,6,11]. Research indicates that parental loss is associated with significant emotional suffering, reflected in high rates of both internalizing symptoms, such as anxiety and depression, and externalizing symptoms, as well as trauma-related manifestations, particularly when the death is sudden or violent [12,13]. In the specific context of orphanhood resulting from femicide, these impacts are often more complex and enduring, affecting overall well-being and psychosocial development and necessitating adaptive reorganizations across various areas of everyday life [3,12].

In this context, health-related quality of life (HRQoL) represents an important outcome for understanding the psychosocial consequences of femicide among orphaned children and adolescents, as it encompasses physical, emotional, social, and functional dimensions of perceived well-being [14,15]. By providing a multidimensional assessment of children’s functioning, HRQoL makes it possible to examine how adverse experiences, including trauma, emotional distress, and social vulnerability, affect different areas of daily life and development [16]. Given the complex and systemic nature of orphanhood resulting from femicide, the perspectives of legal caregivers constitute an important complementary source of information for understanding the psychosocial functioning of affected children and adolescents. This is particularly relevant because caregivers often assume responsibility for the child’s care under circumstances characterized by emotional distress, financial strain, and legal challenges following the loss of the mother [17,18].

Despite growing recognition of the severity of orphanhood resulting from femicide, important gaps remain in the literature. Existing research is largely characterized by qualitative and exploratory approaches or relies on clinical reports, whereas quantitative studies employing standardized measures to assess both mental health and health-related quality of life in this population remain scarce [19]. Moreover, few studies have incorporated the perspectives of both children and adolescents and their legal caregivers, limiting a more comprehensive understanding of the psychosocial consequences of femicide-related orphanhood [20]. In the Brazilian context, these gaps become even more relevant given the high magnitude of femicide and the need to produce evidence capable of supporting interventions and public policies directed at orphans. Specifically, this study adds to the literature by using standardized, validated instruments to jointly assess anxiety, depression, and HRQoL in a sample of children and adolescents orphaned by femicide, and by testing, through mediation analysis, whether depressive symptoms account for the relationship between anxiety and HRQoL an association that has previously been described mainly in qualitative or case-based accounts rather than tested statistically in this specific population.

### Study Aims and Hypotheses

Given this scenario, the present study aimed to investigate health-related quality of life (HRQoL) and its relationship with symptoms of anxiety and depression among children and adolescents orphaned by femicide. Specifically, the study sought to (a) describe levels of HRQoL and internalizing symptoms within the sample; (b) examine the associations between HRQoL, anxiety, and depression; (c) explore differences in these outcomes according to sociodemographic characteristics, particularly gender and age group; and (d) test predictive and mediation models to assess the extent to which anxiety and depressive symptoms contribute to variations in HRQoL. Based on the literature reviewed above, three hypotheses were formulated: (H1) anxiety and depressive symptoms would each show a negative association with HRQoL; (H2) depressive symptoms would be a stronger predictor of HRQoL than anxiety symptoms; and (H3) depressive symptoms would mediate the association between anxiety and HRQoL. Given the cross-sectional design of the study, these hypotheses were tested in terms of statistical association and indirect effects, rather than causal mechanisms.

## 2. Materials and Methods

### 2.1. Study Design

This is a quantitative, cross-sectional study with a correlational and comparative design, conducted with children and adolescents orphaned by femicide residing in the Federal District, Brazil.

### 2.2. Participants and Setting

The sample comprises 57 participants selected through non-probabilistic purposive sampling. Inclusion criteria were: (a) being the child of a femicide victim; (b) being between 8 and 18 years of age; and (c) residing in the Federal District. Data collection was carried out in partnership with the “Acolher Eles e Elas” Program, affiliated with the Secretariat of Justice and Citizenship of the Federal District (SEJUS-DF), which is responsible for monitoring children and adolescents orphaned by femicide. At the time of data collection, the program had registered 203 children and adolescents orphaned by femicide. The program is part of a broader social protection network that offers psychosocial support and institutional benefits to families, thereby facilitating participant identification and contact with participants.

The participants were identified through institutional referrals from the program’s service centers, whose multidisciplinary teams had already established relationships with the families. Recruitment occurred over a two-month period. Children and adolescents who attended their routine follow-up appointments at the program’s service centers were invited to participate. As part of the program’s standard protocol, families attend monthly follow-up appointments during the first six months after enrollment and, subsequently, semiannual appointments. In addition, invitations were extended to families who were not scheduled for appointments during the data collection period, and some agreed to attend the service centers specifically to participate in the study. Data collection took place at these service centers, environments where children, adolescents, and caregivers were already receiving psychological and social support.

Questionnaires were administered by undergraduate psychology students serving as research assistants and by professionals affiliated with the “Acolher Eles e Elas” Program. All assessors received prior training regarding the study protocol, ethical procedures, standardized administration of the instruments, and the management of potential emotional distress during assessment. Because data collection was conducted within the program’s facilities, multidisciplinary professionals, including psychologists and social workers, were immediately available whenever participants experienced emotional discomfort or requested psychological support during or after the assessment.

### 2.3. Measures

#### 2.3.1. Health-Related Quality of Life

Health-related quality of life (HRQoL) was assessed using the KIDSCREEN-27, a widely used measure developed within the framework of the European KIDSCREEN project [14] and later adapted and validated for the Brazilian population [21]. The instrument comprises five dimensions of well-being: physical well-being, psychological well-being, autonomy and parent relations, peers and social support, and school environment. Responses are provided on five-point Likert scales, with response categories varying according to the content of each item. Higher scores reflect a more favorable perception of HRQoL. To illustrate the content of each dimension, items ask, in general terms, whether the child has felt physically fit and energetic (Physical Well-Being), whether the child has felt happy and content with life (Psychological Well-Being), whether the child has felt supported and able to talk with a parent or guardian about personal matters (Autonomy and Parent Relations), whether the child has had opportunities to spend time with and be helped by friends (Peers and Social Support), and whether the child has felt capable of paying attention and getting along at school (School Environment). Preliminary reliability analyses indicated that two items from the Autonomy and Parent Relations dimension of the KIDSCREEN-27—Item 6 (“Have you had enough money to do the same things as your friends?”) and Item 7 (“Have you had enough money for your expenses?”)—presented low item-total correlations and substantially reduced the internal consistency of the dimension (α = 0.59). After removing these items, internal consistency increased to an acceptable level (α = 0.71). Although the KIDSCREEN-27 is a validated instrument, these items may have been less applicable or less meaningful within the socioeconomic characteristics of the present sample, or may have been interpreted differently by participants, which could explain their poor psychometric performance. Therefore, the decision to exclude these items was made exclusively to improve the reliability of this specific dimension while preserving the remaining structure of the instrument.

#### 2.3.2. Anxiety and Depressive Symptoms

Symptoms of anxiety and depression were assessed using the short version of the Revised Child Anxiety and Depression Scale (RCADS-25-Y), a widely used instrument for screening internalizing symptoms in children and adolescents [22]. The Brazilian Portuguese version used in this study was obtained from the official UCLA Child FIRST RCADS repository and has been previously adapted for use in Brazil [23]. The scale comprises 25 items distributed across two domains—anxiety and depression—and is rated on a four-point Likert scale. Higher scores indicate a greater frequency of emotional symptoms. To illustrate item content, the anxiety subscale includes statements about worrying about things going wrong or feeling afraid in specific situations, whereas the depression subscale includes statements about feeling sad, having little energy, or losing interest in activities that used to be enjoyable. In the present study, the RCADS-25-Y demonstrated excellent internal consistency for the total scale (α = 0.883), as well as good reliability for the depression (α = 0.796) and anxiety (α = 0.825) subscales.

#### 2.3.3. Sociodemographic Questionnaire

A sociodemographic questionnaire was used to gather information on participants’ age, sex, relationship to their legal guardian, family income, educational attainment, and time elapsed since the femicide. Given the sensitive nature of the study population, direct questions about the femicide were intentionally omitted to minimize psychological distress and reduce the risk of emotional reactivation associated with the traumatic event. Information concerning the year of the occurrence was subsequently retrieved from institutional records. The “time since event” variable was computed as the difference, in whole years, between the year of data collection and the year the femicide occurred (e.g., a value of 0 indicates that the femicide occurred in the same calendar year as data collection); this variable was therefore based on institutional records rather than participant report.

### 2.4. Data Collection Procedures

Potential participants were first approached by psychologists from the support centers who were already involved in monitoring and supporting the families. After being informed about the aims and procedures of the study, legal guardians and children/adolescents were invited to take part in the research.

Data were collected individually by six trained Psychology students, with support from professionals working at the centers. The questionnaires were administered in a self-report format in private rooms to ensure confidentiality and facilitate participants’ engagement. Administrators remained available to answer questions and provide clarification as needed. Informed consent from legal guardians and assent from children and adolescents were obtained before data collection. Completion of the assessment protocol required approximately 10 to 20 min.

### 2.5. Ethical Considerations

The study was approved by the Research Ethics Committee (Approval No. 7.674.519). Participation was voluntary and adhered to ethical principles of confidentiality, privacy, and the protection of vulnerable populations. Due to the sensitive nature of the subject matter and the possibility of emotional distress, data collection was conducted within the support centers where children, adolescents, and their families were already receiving psychosocial services. In situations of emotional distress identified during the application, participants could be promptly supported and referred to specialized care.

### 2.6. Statistical Analysis

Statistical analyses were conducted using IBM SPSS Statistics version 25. Initially, descriptive statistics (means, standard deviations, and frequencies) were calculated to characterize the sample and the variables under investigation. Data distribution was assessed using the Shapiro–Wilk and Kolmogorov–Smirnov tests, which informed the selection of parametric or non-parametric statistical procedures. The full sample comprised 57 participants; minor variation in the degrees of freedom across analyses (e.g., N = 55 for the RCADS-25 subscales, N = 56 for the School Environment dimension) reflects item-level missing data on those specific measures. Missing data were handled using listwise deletion, applied separately within each analysis rather than as a single global exclusion for the whole study.

Associations between continuous variables were examined using Spearman’s rank-order correlations. Group comparisons were performed using Student’s *t*-tests or Mann–Whitney *U* tests, depending on whether the assumptions for parametric analyses were met. Given the non-normal distribution of the data, predictive analyses were conducted using linear regression models with bootstrap resampling (1000 samples), providing more robust parameter estimates. To further strengthen the analytical rigor of the study, Generalized Linear Models (GLMs) were also estimated. This complementary approach allowed for inferences that were less sensitive to violations of the normality assumption, thereby enhancing the robustness and reliability of the findings. Mediation and moderation models were estimated using the PROCESS macro v4.2 for SPSS [24] with 5000 bootstrap resamples and 95% percentile confidence intervals. Given the exploratory nature of the study and the number of correlations and group comparisons conducted, no correction for multiple comparisons (e.g., Bonferroni) was applied; consequently, findings should be interpreted with appropriate caution regarding the risk of Type I error, and are considered hypothesis-generating rather than confirmatory. No a priori power analysis was conducted; given the modest sample size (N = 57 overall, reduced to N = 54 for the mediation models due to listwise deletion of missing data), the study may have been underpowered to detect small effects, particularly in the regression, mediation, and moderation models, and this is acknowledged as a limitation.

## 3. Results

This section presents the results of the analyses examining health-related quality of life (HRQoL) and mental health outcomes, specifically symptoms of anxiety and depression, in children and adolescents orphaned by femicide. The sociodemographic characteristics of the participants are presented in Table 1.

The study sample comprised 57 children and adolescents whose mothers had been victims of femicide in the Federal District. Regarding gender, 52.6% (N = 30) were girls and 43.9% (N = 25) were boys. In terms of self-identified race/color, most participants identified as mixed-race (56.1%), followed by White (21.1%) and Black (17.5%). With respect to education level, the majority had completed primary education (70.2%), while 28.1% reported having completed secondary education. Most participants were students (89.5%). Regarding religious affiliation, Evangelical (38.6%) and Catholic (36.8%) denominations were the most frequently reported.

Participants had a mean age of 12.55 years (SD = 3.47), ranging from 8 to 18 years. The average household size was 4.25 individuals (SD = 2.44). The mean time elapsed since the femicide was five years, with values ranging from 0 years—indicating that the event occurred in the same year as data collection—to 10 years. Descriptive statistics for the continuous variables are presented in Table 2.

The results of the KIDSCREEN-27 (scores ranging from 0 to 100) indicated a mean health-related quality of life (HRQoL) score of 70.70 (SD = 14.66). Among the assessed dimensions, Social Support and Friends showed the highest mean score (M = 76.10, SD = 19.70), followed by Autonomy and Parent Relations (M = 74.95, SD = 20.00). In contrast, Physical Well-Being presented the lowest mean score (M = 65.48, SD = 16.92), as shown in Table 2. Mental health was assessed using the RCADS-25, which yielded a mean total score of 10.30 (SD = 5.84). Analysis of the subscales revealed higher scores for Anxiety (M = 12.79, SD = 7.36) than for Depression (M = 7.81, SD = 5.13).

The normality of the distributions was assessed using the *Kolmogorov–Smirnov* and *Shapiro–Wilk tests*, with the latter being prioritized due to the sample size. The results indicated a violation of the normality assumption for all dimensions of the KIDSCREEN-27 and RCADS-25 (*p* < 0.05), indicating non-normal distributions. For analysis purposes, the Adolescent variable was coded as 1 (13 to 18 years) and Child was coded as 0 (8 to 12 years). The Gender variable was coded as 1 (female) and 0 (male).

Table 3 presents the Spearman correlations between the RCADS-25 and KIDSCREEN-27 dimensions and the sociodemographic variables age group, gender, and time since the femicide. Age group was negatively associated with health-related quality of life (HRQoL), as evidenced by significant correlations with the total KIDSCREEN-27 score (r = −0.375, *p* < 0.05) and with the dimensions of Physical Well-Being(r = −0.406, *p* < 0.05), Psychological Well-Being (r = −0.351, *p* < 0.05), Autonomy and Parent Relations (r = −0.319, *p* < 0.05), and School Environment (r = −0.343 *p* < 0.05). Gender was significantly associated with mental health outcomes. Positive correlations were observed with RCADS-25 depression (r = 0.297, *p* < 0.05), anxiety (r = 0.287, *p* < 0.05), and total symptom scores (r = 0.313, *p* < 0.05), indicating higher levels of emotional symptoms among one gender category. In contrast, gender was negatively correlated with the KIDSCREEN-27 dimensions of physical health (r = −0.288, *p* < 0.05) and Psychological well-being (r = −0.294, *p* < 0.05), suggesting lower perceived quality of life in these domains.

No statistically significant correlations were found between time since the femicide and any of the mental health or quality-of-life measures (*p* > 0.05). This finding indicates that, in the present sample, the time elapsed since the event was not associated with differences in psychological symptoms or perceived quality of life. These results suggest that older participants reported lower levels of quality of life across several domains.

Table 4 presents the results of the gender-based comparisons in health-related quality of life and mental health dimensions, conducted using the Mann–Whitney *U* test. On the KIDSCREEN-27, boys scored significantly higher than girls in the Physical Well-Being dimension (M = 70.15; *U* = 249; *p* = 0.052; d = 0.603). A similar pattern was observed for Emotional State, with boys reporting significantly better outcomes than girls (*U* = 244; *p* = 0.044; d = 0.589). No statistically significant gender differences were found in the remaining KIDSCREEN-27 dimensions—Autonomy and Parent Relations, Social Support and Friends, and School Environment—or in the overall quality of life score (*p* > 0.05).

In contrast, the RCADS-25 results indicated poorer mental health outcomes among girls. Female participants reported significantly higher levels of depressive symptoms (M = 15.16; SD = 7.76) than male participants (M = 6.18; SD = 3.49), with the difference reaching statistical significance (*U* = 217; *p* < 0.05; d = −0.758). A similar pattern was observed for anxiety symptoms, with girls obtaining a higher mean score (M = 15.17; SD = 7.77) than boys (M = 10.75; SD = 6.13), also yielding a significant difference (*U* = 211; *p* < 0.05; d = −0.715). This gender disparity was further reflected in the total RCADS-25 score, for which girls showed significantly higher overall levels of emotional symptoms than boys (*U* = 204; *p* < 0.05; d = −0.795).

Table 5 presents the comparisons between children and adolescents across the dimensions of health-related quality of life. Children reported significantly higher scores than adolescents in the Health and Physical Activity dimension (M = 70.34, SD = 14.88 vs. M = 59.90, SD = 16.77), a difference confirmed by the Mann–Whitney *U* test (*U* = 217, *p* < 0.05). A similar pattern emerged for Emotional State, with children reporting greater well-being (M = 77.46) than adolescents (M = 65.18), accompanied by a moderate-to-large effect size (d = 0.714, *p* < 0.05). Significant age-group differences were also observed in the Family and Leisure Time dimension, with higher scores among children (M = 51.35) compared to adolescents (M = 41.96) (d = 0.681, *p* < 0.05). In addition, the total KIDSCREEN-27 score differed significantly between groups according to the Mann–Whitney *U* test (*U* = 234, *p* = 0.043), indicating a more favorable overall perception of quality of life among children. No significant differences were found between children and adolescents in the Social Support and Friends or School Environment dimensions (*p* > 0.05).

Table 6 and Table 7 present the results of a multiple linear regression analysis with Bootstrap estimation conducted to examine the combined effects of anxiety, depression, and age group on health-related quality of life. The overall model was statistically significant and explained 47% of the variance in the outcome variable [F(3, 50) = 16.404, *p* < 0.001]. As shown in Table 7, depression emerged as the strongest predictor of health-related quality of life (β = −2.541, *p* < 0.001), indicating that higher levels of depressive symptoms were associated with poorer quality of life. In contrast, anxiety did not make a significant unique contribution to the model after controlling depression and age group (β = 0.620, *p* > 0.05). Likewise, age group was not a significant predictor of health-related quality of life (*p* > 0.05).

Table 8 presents Bootstrap Linear Regression and the Generalized Linear Model (GLM) lends robustness to the findings, indicating that depression is the predominant negative predictor of HRQoL in this sample (*p* < 0.001). In both models, anxiety, when controlled for depression, assumes a positive and significant coefficient (*p* < 0.05), signaling a phenomenon of statistical suppression. The systematic maintenance of this sign reversal in different estimation methods suggests that the relationship between anxiety and HRQoL is not direct but rather processed by the depression variable. Therefore, a mediation model was tested using *path analysis*, in which depression acted as a mediating variable in the relationship between anxiety and health-related quality of life.

**Figure 1:**
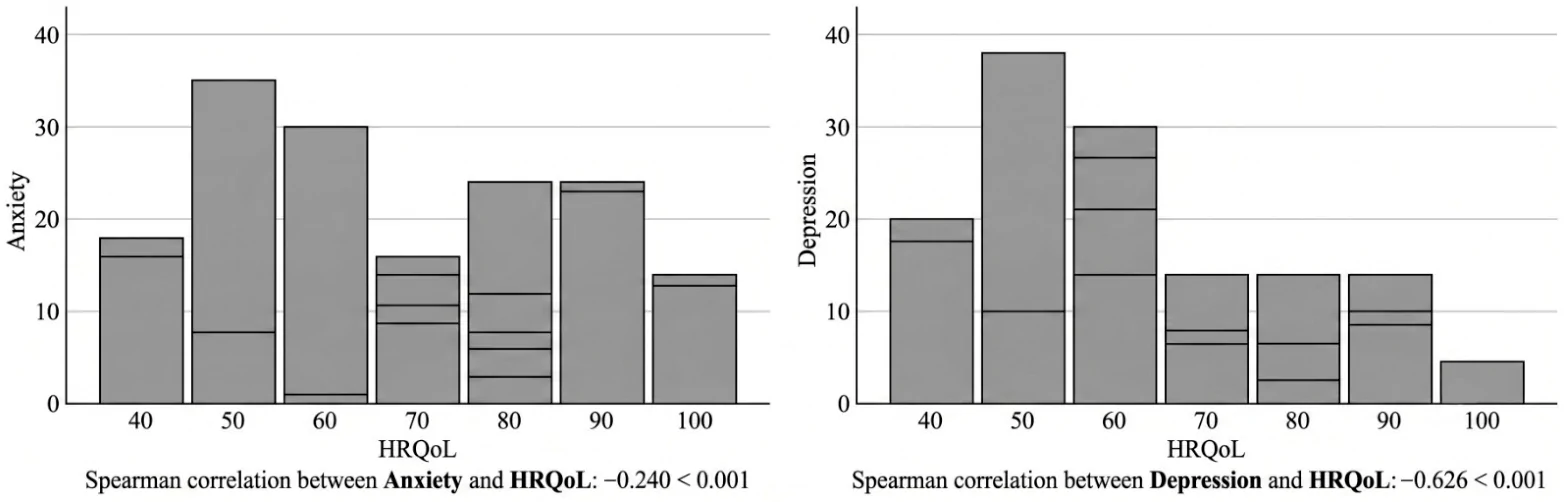
Levels of Anxiety and Depression by Quality of Life Strata (QOL). Note: HRQoL strata on the x-axis correspond to ranges of the KIDSCREEN-27 total score (grouped in bands of approximately 10 points, e.g., 40–49, 50–59, etc.); the y-axis shows RCADS-25 anxiety or depression scores for participants within each band.

Figure 1 illustrates the distribution of anxiety and depression scores in relation to health-related quality of life and their respective correlations. Depression exhibited a substantially stronger negative association with quality of life (r = −0.626, *p* < 0.001) than anxiety (r = −0.240, *p* < 0.001). In the initial ranges, the anxiety and depression scores show similar intensities. However, from a QoLRS score of 70 onwards, the pattern changes: anxiety remains high, while depression decreases.

**Figure 2:**
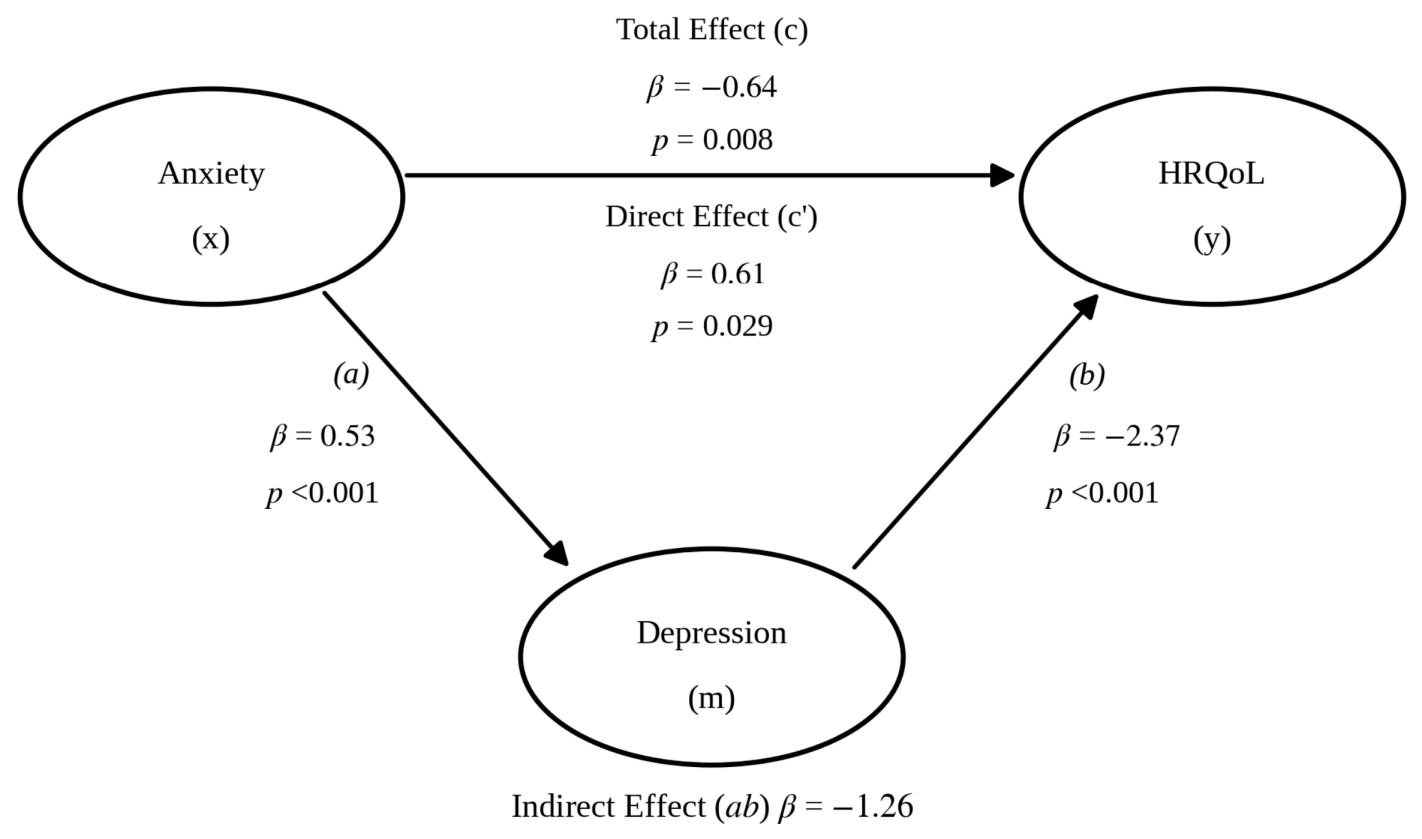
Representation of the Mediation Model between Anxiety, Depression, and Health-Related Quality of Life (HRQoL).

Table 9 summarizes the overall fit of the mediation model, which was statistically significant. Furthermore, Table 10 and Figure 2 present the results of the mediation analysis, indicating that depression mediates the relationship between anxiety and health-related quality of life (HRQoL). Anxiety was positively associated with depression (β = 0.53, *p* < 0.001), while depression, in turn, was negatively associated with HRQoL (β = −2.37, *p* < 0.001).

**Figure 3:**
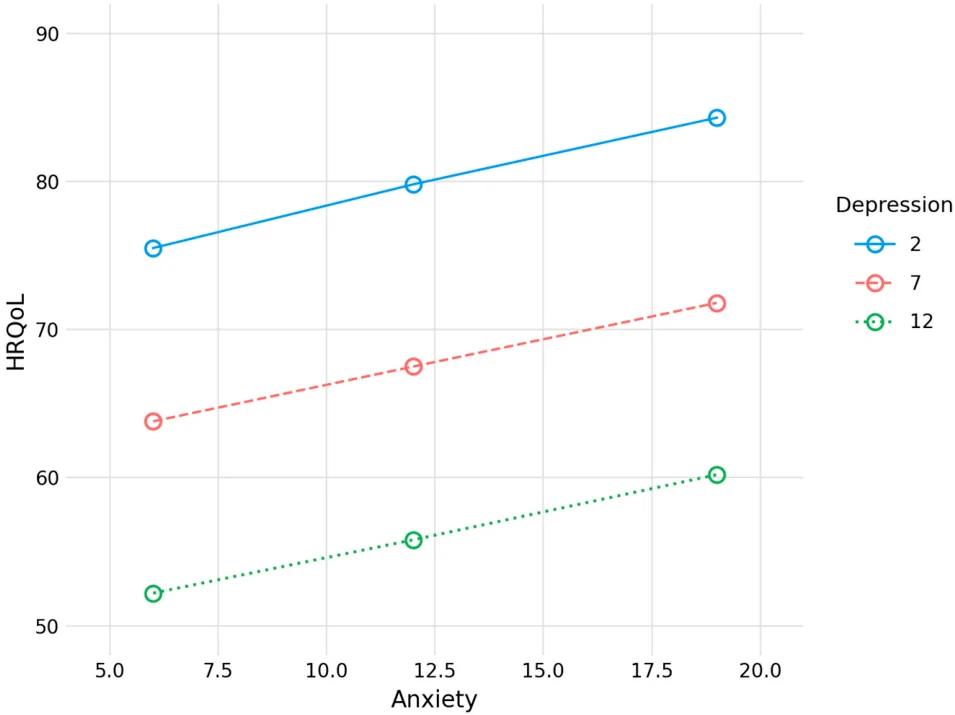
Analysis of the Moderation of Depression in the Relationship between Anxiety and Health-Related Quality of Life (HRQoL).

Table 11 presents the results of the moderation analysis conducted to examine whether the association between anxiety and health-related quality of life (HRQoL) varied according to levels of depressive symptoms. The overall regression model was statistically significant and explained 49% of the variance in HRQoL [F(3, 50) = 16.49, *p* < 0.001]. However, the interaction term between anxiety and depression was not statistically significant (*β* = −0.003; *p* > 0.05), indicating the absence of a moderating effect. This interpretation is further supported by Figure 3, which shows a similar pattern in the association between anxiety and HRQoL regardless of the level of depressive symptoms. Together, these findings suggest that anxiety and depression contribute independently to variations in HRQoL. While depression plays a central role in predicting poorer quality of life, it does not alter the magnitude of the association between anxiety and HRQoL.

## 4. Discussion

Contrary to the intuitive expectation that the passage of time may function as a protective factor for mental health following a traumatic event, the results of the present study did not reveal significant associations between time elapsed since the femicide and any of the mental health or health-related quality of life dimensions assessed (Table 3). This finding suggests that psychological adjustment following such a traumatic loss may not occur as a simple function of time. Previous research has shown that children exposed to parental intimate partner homicide may experience long-lasting psychological consequences, and that their adaptation is influenced by multiple individual, familial, and contextual factors rather than by the passage of time alone [10,20]. In this context, chronological time alone appears insufficient to explain adaptation to traumatic events. Rather, recovery is likely influenced by a range of contextual and psychosocial factors, including the quality of social support, family stability, access to mental health services, and exposure to cumulative adversities [1,20,25].

The results indicated that age showed moderate negative correlations with the total score of the KIDSCREEN-27 and its dimensions, suggesting that older adolescents perceive lower levels of physical, emotional, family, and school well-being (see Table 3). These findings suggest that increasing age is associated with a lower perceived quality of life and well-being among the participants. Consistent with these results, studies using the KIDSCREEN instrument have shown that older adolescents tend to report poorer health-related quality of life across multiple domains compared to younger adolescents. [26].

Unlike time since the femicide, gender emerged as a significant correlate of mental health outcomes, particularly symptoms of depression and anxiety measured by the RCADS-25 (Table 3). These findings are consistent with previous research showing that girls are more likely than boys to experience internalizing difficulties, especially during adolescence. According to Leach et al. [27], psychosocial factors may contribute to the greater vulnerability of females to anxiety disorders. In the present study, this pattern was reflected in the significant positive associations between gender and both anxiety and depression scores (*p* < 0.05). Longitudinal evidence indicates that adverse childhood experiences, such as exposure to interpersonal violence, are associated with higher risks of depression and anxiety throughout development, with more pronounced effects in females [28]. In addition, studies with children and adolescents show that girls exposed to violence exhibit higher levels of anxiety and depressive symptoms compared to boys, possibly due to differences in emotional socialization processes, greater internalization of suffering, and greater sensitivity to interpersonal stressors [29]. These findings help understand why, in the present study, gender was more significantly associated with dimensions of mental health.

In contexts of violent loss, such as femicide, depression frequently emerges as a central outcome of traumatic grief, especially when the child or adolescent has been previously exposed to domestic violence [1,13]. Recent reviews have shown that severe adverse experiences during childhood, including the homicide of a caregiver, are strongly associated with elevated levels of depression that may persist throughout development [28,29]. Thus, the model shows that in this population, depression is not only an associated symptom but a key mechanism in the deterioration of HRQoL.

The absence of a statistically significant effect of anxiety in the regression model is consistent with previous research indicating that, although anxiety and depression commonly co-occur among individuals exposed to violence, depressive symptoms tend to exert a stronger influence on health-related quality of life (HRQoL) [30,31]. In multivariate models, depression can absorb some of the variance shared with anxiety, especially in contexts of chronic suffering and irreversible losses, as observed in orphans of femicide [3,4]. Thus, the absence of significance does not indicate clinical relevance of anxiety but suggests that its impact on HRQoL may be mediated or overlapped by depressive symptoms.

The graphs displayed in Figure 1 support the findings of the regression model presented. It is observed that while depressive symptoms decrease as HRQoL scores reach higher levels, anxiety remains present in a higher and more unstable form, suggesting that anxious suffering can persist even in orphans with good HRQoL perception. Previous research suggests that anxiety may persist due to enduring states of hypervigilance and dispositional characteristics, such as neuroticism, whereas depression is more strongly characterized by feelings of helplessness, hopelessness, and diminished meaning in life [27]. The presence of anxiety does not always imply a worse assessment of quality of life, especially when there is essential support for these people [8,29].

The results of the mediation analysis (Table 10) indicate that the statistical association between anxiety and health-related quality of life (HRQoL) is largely accounted for by depressive symptoms. It is important to note that anxiety, depression, and HRQoL were all measured concurrently in this cross-sectional design; the mediation model therefore describes a statistical pattern of shared variance consistent with depression as an intervening variable, rather than establishing a causal or temporal sequence in which anxiety produces depression, which in turn produces poorer HRQoL. Although anxiety is associated with HRQoL, its indirect effect through depression was substantially stronger, which is consistent with the broader literature pointing to depression as a central psychological correlate of quality-of-life deterioration in children and adolescents exposed to violent losses. The literature on traumatic grief and violent losses supports this functional distinction between anxiety and depression. Kristensen et al. [13] reported that, following sudden and violent losses, anxiety symptoms are often more prominent during the initial phases of adaptation, whereas depressive symptoms tend to persist over time and are more strongly associated with functional impairment and reduced quality of life. Qualitative research and integrative reviews indicate that victims of femicide frequently experience abrupt ruptures in affective bonds, instability in care arrangements, and a feeling of institutional abandonment, factors that favor depressive trajectories more than isolated anxiety disorders [1,3,4,17].

The absence of a moderating effect of depression on the relationship between anxiety and HRQoL is consistent with this interpretation (see Table 11). The results suggest that depression does not alter the strength or direction of the statistical association between anxiety and HRQoL, but is better characterized, in this cross-sectional model, as an intervening variable that carries part of the shared association between anxiety and HRQoL, rather than as a moderator. In the context of violent losses, and pending confirmation from longitudinal designs, support focused on reducing depressive symptoms may be clinically relevant, as it could plausibly help mitigate the impairment that anxiety is associated with in quality of life [15,16].

In the context of femicide orphans, experiencing the process culminating in the mother’s death and the post-mortem period exposes them to abrupt ruptures, maternal loss, broken community ties, family disorganization, and social stigmatization, which can compromise their subjective perception of well-being [15]. Depression and anxiety function as interconnected risk factors that affect not only emotional well-being but also social relationships, school engagement, and developmental outcomes across the life span [15]. Interventions aimed at reducing apathy, isolation, and hopelessness associated with depression tend to produce indirect effects on anxiety management and HRQoL. Given the central role of depression identified in the present study, interventions aimed at reducing symptoms such as apathy, social withdrawal, hopelessness, and loss of interest may have broader benefits that extend beyond depressive symptomatology itself. Evidence suggest that improvements in depressive symptoms can indirectly contribute to better anxiety management and enhanced health-related quality of life (HRQoL), even when they do not directly address anxiety symptoms [3]. Furthermore, the role of caregivers and the support network is particularly important, as they provide predictability, emotional security, and the necessary support to process grief and facilitate the reconstruction of developmental trajectories that are less affected by functional impairment [17,18].

### 4.1. Limitations

This study has some limitations that should be considered when interpreting the findings. First, the cross-sectional design does not allow causal or temporal conclusions to be drawn: anxiety, depression, and HRQoL were measured concurrently, and the mediation and moderation models describe patterns of shared statistical association rather than causal pathways. Longitudinal designs are needed to examine how these variables unfold over time following femicide-related orphanhood.

Second, the sample was recruited through non-probabilistic purposive sampling from a single institutional program in the Federal District of Brazil, which limits the generalizability of the findings to other regions or contexts. The sample size (N = 57) may also have limited statistical power to detect small effects, particularly in the regression, mediation, and moderation models.

Finally, the study did not include specific measures of posttraumatic stress, prolonged or traumatic grief, prior exposure to domestic violence, witnessing status at the time of the femicide, relationship to the perpetrator, or the stability of current caregiving arrangements. Depressive symptoms in this population may partly overlap with traumatic bereavement and complex trauma reactions rather than reflecting a depressive disorder dimension alone [11,13,18]; future studies should assess these constructs directly to disentangle their relative contributions to HRQoL.

### 4.2. Final Considerations

This study found that health-related quality of life (HRQoL) in children and adolescents orphaned by femicide is closely and negatively associated with depressive symptoms, which emerged as the strongest negative predictor of HRQoL among the variables examined. The findings also indicated that depression is statistically associated with, and may partly account for, the relationship between anxiety and HRQoL, consistent with the negative association of anxiety on quality of life operating largely alongside elevated depressive symptoms. Given the cross-sectional design, this pattern should be interpreted as an indirect statistical association rather than a demonstrated causal mechanism. These results are nonetheless consistent with evidence suggesting that, in the aftermath of violent loss, depression may play a central role in the deterioration of psychosocial functioning [13].

The study also identified significant gender differences in mental health outcomes. Girls presented higher levels of depression and anxiety, as well as poorer scores in the emotional dimensions of HRQoL. Moreover, HRQoL scores tended to decrease with age, particularly among adolescents. This pattern suggests that the increasing developmental demands of adolescence, together with the persistent consequences of femicide-related orphanhood, may contribute to greater impairment in well-being over time [26].

From a theoretical perspective, the present study contributes to the growing body of research that conceptualizes health-related quality of life (HRQoL) as a multidimensional construct influenced by the interplay of emotional, developmental, and contextual factors. By showing, through mediation analysis, that depressive symptoms statistically account for much of the association between anxiety and HRQoL, the findings offer preliminary insight into the psychological processes that may be involved in the well-being of children and adolescents affected by femicide-related orphanhood, while underscoring the need for longitudinal research to confirm the direction of these associations. Moreover, the study expands the limited empirical literature on the consequences of femicide for surviving children, a population that remains underrepresented in quantitative research in the Brazilian context.

From a practical and institutional perspective, the results underscore the importance of public policies and psychosocial interventions aimed at the early detection and treatment of depressive symptoms among children and adolescents affected by femicide. Care strategies that consider gender specificities, developmental stage, and the strengthening of family and community support networks are fundamental for promoting mental health and quality of life in this population. Interventions focused on reducing isolation, hopelessness, and emotional disorganization associated with depression tend to produce positive indirect effects on anxiety and overall well-being, supporting developmental trajectories characterized by greater well-being and resilience.

## Figures and Tables

**Table 1:** Sociodemographic profile.

Variables	N	%
Gender		
Female	30	52.6
Male	25	43.9
Did not answer	2	3.5
Race or Ethnicity		
Asian	2	3.5
White	12	21.1
Mixed-race	32	56.1
Black	10	17.5
Did not answer	1	1.8
Educational Attainment		
Elementary School	40	70.2
High School	16	28.1
Did not answer	1	1.8
Occupation		
Unemployed	2	3.5
Student	51	89.5
Informal employment	2	3.5
Did not answer	2	3.5
Religion		
Catholic	21	36.8
Spiritist	1	1.8
Evangelical	22	38.6
No religion	9	15.8
Other religion	1	1.8
Did not answer	3	5.3

**Table 2:** Description of scores from the KIDSCREEN-27 and RCADS-25 scales.

Measures	N	Minimum	Maximum	Mean	SD
KIDSCREEN-27					
Physical Well-being	57	25.00	95.00	65.48	16.92
Psychological Well-being	57	21.43	100.00	72.49	17.73
Autonomy and Parent Relations	57	25.00	100.00	74.95	20.00
Social Support and Peers	57	12.50	100.00	76.10	19.70
School Environment	56	18.75	100.00	72.10	19.18
Total Score	57	31.69	97.89	70.70	14.66
RCADS-25					
Depression	55	0.00	24.00	7.81	5.13
Anxiety	55	1.00	35.00	12.79	7.36
Total Score	55	1.00	29.50	10.30	5.84

**Table 3:** Spearman Correlation.

		RCADS-25 Dimensions			KIDSCREEN-27 Dimensions					
		Depression	Anxiety	RCADS-25 (Total Score)	Physical Well-Being	Psychological Well-Being	Autonomy and Parent Relations	Social Support	School Environment	KIDSCREEN-27 (Total Score)
Age Group	R	0.177	−0.21	−0.048	−0.406 **	−0.351 **	−0.319 *	0.025	−0.343 *	−0.375 **
	Sig.	0.199	0.128	0.733	0.002	0.008	0.016	0.857	0.01	0.004
Gender	R	0.297 *	0.287 *	0.313 *	−0.288 *	−0.294 *	−0.133	−0.028	0.047	−0.19
	Sig.	0.031	0.037	0.022	0.033	0.029	0.332	0.84	0.737	0.165
Time since event	R	0.115	0.135	0.102	0.017	−0.058	−0.199	−0.031	0.071	−0.097
	Sig.	0.443	0.365	0.494	0.907	0.69	0.17	0.832	0.632	0.508

* Correlation is significant at the 0.05 level (2-tailed); ** Correlation is significant at the 0.01 level (2-tailed).

**Table 4:** Comparison between genders in the dimensions of the KIDSCREEN and RCADS-25 using the Mann–Whitney test.

Scales	Gender	N	Mean	SD	*U*	*p*	d de Cohen
Physical Well-Being	Female	30	59.9583	17.3593	249	0.052	0.603
	Male	25	70.15	13.80821			
Psychological Well-Being	Female	30	67.0238	19.99919	244	0.044	0.589
	Male	25	77.5714	12.43692			
Autonomy and Parent Relations	Female	30	71.8333	23.17413	296	0.262	0.433
	Male	25	79.4857	14.95248			
Social Support and Peers	Female	30	75.2083	21.30465	354	0.916	0.110
	Male	25	77.75	16.34587			
School Environment	Female	29	73.0603	18.68317	318	0.589	−0.168
	Male	25	70.75	20.46974			
KIDSCREEN (T-SCORE)	Female	30	67.6845	16.83441	289	0.216	0.388
	Male	25	73.8331	11.49275			
Depression	Female	28	9.7143	5.70286	217	0.029	−0.758
	Male	25	6.1778	3.49779			
Anxiety	Female	28	15.1684	7.76644	211	0.022	−0.715
	Male	25	10.7514	6.13479			
RCADS	Female	28	12.4413	6.26669	204	0.015	−0.795
	Male	25	8.4646	4.4027			

**Table 5:** Comparison between age groups in the dimensions of the KIDSCREEN and RCADS-25 using the Mann–Whitney *U* test.

Scales	Age group	N	Mean	SD	*U*	*p*	d de Cohen
Physical Well-Being	Child	29	70.34	14.88	217	0.018	0.6631
	Adolescent	24	59.90	16.77			
Psychological Well-Being	Child	29	77.46	16.15	206	0.011	0.7143
	Adolescent	24	65.18	18.4			
Autonomy and Parent Relations	Child	29	51.35	12.31	226	0.028	0.6805
	Adolescent	24	41.96	15.42			
Social Support and Peers	Child	29	73.71	20.14	301	0.401	−0.2389
	Adolescent	24	78.39	18.88			
School Environment	Child	29	75.00	21.84	247	0.07	0.3127
	Adolescent	24	69.01	15.25			
KIDSCREEN (T-SCORE)	Child	29	69.57	12.87	234	0.043	0.5206
	Adolescent	24	62.89	12.81			
Depression	Child	27	7.02	4.45	259	0.219	−0.3689
	Adolescent	24	8.95	5.97			
Anxiety	Child	27	13.96	7.54	266	0.273	0.3219
	Adolescent	24	11.57	7.25			
RCADS	Child	27	20.98	11.19	319	0.925	0.0383
	Adolescent	24	20.52	12.84			

**Table 6:** Linear Regression with Bootstrap.

R	R^2^	Adjusted R^2^	Std. Error of the Estimate	R^2^ Change	F Change	df1	df2	Sig. F Change
0.704	0.496	0.466	10.95071	0.496	16.404	3	50	<0.001

**Table 7:** Linear Regression Model with Bootstrap.

		Bootstrap				
					95% Confidence Interval	
Model	B	Bias	Error	Sig.	Lower	Upper
(Constant)	83.742	−0.003	3.345	0.001	77.041	90.259
Depression	−2.541	−0.046	0.553	0.001	−3.699	−1.56
Anxiety	0.62	0.028	0.39	0.12	−0.067	1.449
Age Group	−2.491	−0.065	3.659	0.51	−9.87	4.721

Dependent Variable: HRQoL (KIDSCREEN-27).

**Table 8:** Parameter Estimates of the Generalized Linear Model for Health-Related Quality of Life (HRQoL).

			95% CI for B				
Variables	B	Std. Error	Lower	Upper	Wald χ2	df	Sig.
(Intercept)	81.251	3.2942	74.795	87.708	608.352	1	<0.001
Depression	−2.541	0.4752	−3.472	−1.61	28.593	1	<0.001
Anxiety	0.62	0.3308	−0.028	1.269	3.517	1	0.061
Age Group	2.491	3.3187	−4.014	8.995	0.563	1	0.453

Dependent Variable: HRQoL (KIDSCREEN-27).

**Table 9:** Mediation model between Anxiety, Depression and Health-Related Quality of Life (HRQoL).

R	R-Sq	MSE	F	df1	df2	*p*
0.7052	0.4973	93.0403	25.2288	2	51	<0.001

**Table 10:** Mediation between Anxiety, Depression and Health-Related Quality of Life (HRQoL).

Path	Coefficient (B)	Std. Error (SE)	*t*	*p*	95% CI
Anxiety—Depression (a)	0.53	0.06	8.15	<0.001	[0.40, 0.66]
Depression—Quality of Life (b)	−2.37	0.38	−6.14	<0.001	[−3.15, −1.59]
Direct Effect (c’)	0.61	0.27	2.24	0.029	[0.06, 1.16]
Total Effect (c)	−0.64	0.23	−2.72	0.008	[−1.12, −0.17]
Indirect Effect (ab)	−1.26	0.28	-	-	[−1.89, −0.79]

a = path coefficient for the effect of Anxiety on Depression; b = path coefficient for the effect of Depression on Health-Related Quality of Life (HRQoL), controlling for Anxiety; c′ = direct effect of Anxiety on HRQoL, controlling for Depression; c = total effect of Anxiety on HRQoL; ab = indirect effect of Anxiety on HRQoL through Depression.

**Table 11:** Analysis of the Moderation of Depression in the Relationship between Anxiety and Health-Related Quality of Life (HRQoL).

Variable	B	SE	*t*	*p*	95% IC
Constant	78.39	3.74	20.95	<0.001	[70.87, 85.91]
Anxiety	0.62	0.28	2.21	0.031	[0.05, 1.18]
Depression	−2.36	0.4	−5.9	<0.001	[−3.17, −1.56]
Interaction	−0.003	0.02	−0.11	0.906	[−0.05, 0.05]

## Data Availability

The data are not publicly available due to ethical and privacy restrictions involving a vulnerable population of children and adolescents orphaned by femicide.
